# NeuroBehcet’s-related intracranial hypertension without cerebral venous thrombosis: case report and review of literature

**DOI:** 10.1186/s12883-024-03708-x

**Published:** 2024-06-14

**Authors:** Jinesh Mukesh Shah, Warren Fong, Deidre Anne De Silva

**Affiliations:** 1 Department of Neurology, National Neuroscience Institute, Singapore General Hospital Campus, Singapore, Singapore; 2 Department of Rheumatology and Immunology, Singapore General Hospital, Singapore, Singapore

**Keywords:** Behcet’s disease, NeuroBehcet’s, Intracranial pressure, Cerebral venous thrombosis, Idiopathic intracranial hypertension, Tocilizumab

## Abstract

**Background:**

We present a rare case of NeuroBehcet’s-related intracranial hypertension without cerebral venous thrombosis (NBrIHwCVT), occurring as the first presentation of NeuroBehcet’s. In addition, we describe the novel use of subcutaneous tocilizumab for this indication. This is followed by a review of the literature on this topic.

**Case:**

The patient was a 28-year-old lady of Southern Chinese origin with a known history of Behcet’s disease with oral ulcers and ocular findings for which she was on mycophenolate mofetil and adalimumab. She presented with a headache and bilateral disc swelling associated with an intracranial pressure (ICP) of > 40cmH20. There were no structural lesions or cerebral venous thrombosis (CVT) on imaging. Initial lumbar puncture had raised leucocytes and protein. We discuss diagnostic challenges given persistently elevated ICP despite subsequent non-inflammatory cerebrospinal fluid (CSF) profiles and non-response to acetazolamide. She eventually showed a response to immunosuppressant therapy in the form of pulsed methylprednisolone, cyclophosphamide and subsequently subcutaneous tocilizumab, supporting the diagnosis of NBrIHwCVT. Complete normalization of ICP remains challenging. Her disease course was severe, unusual for her ethnicity.

**Literature review:**

We identified 34 patients (including ours) from 14 publications. We found that the majority of NBrIHwCVT patients were young (average age of 34 years), with a slight female preponderance. Of the 17 cases in the literature with available data on CSF profile, none had raised leucocytes whilst one patient had elevated protein. Patients were generally treated with steroids and occasionally azathioprine, in line with the suspected autoimmune pathophysiology. Of 22 patients with data on outcome, six (27%) were noted to have recurrence of symptoms generally occurring a few months later.

**Conclusion:**

As demonstrated by this case, NBrIHwCVT can present with BD with raised ICP even if there is no prior history of NB, central Asian ethnicity, cerebral venous thrombosis or features of inflammation on the CSF. We demonstrated how novel use of Tocilizumab may have a role in the management of NBrIHwCVT. Based on our literature review, patients were more likely to be young, female, display a non-inflammatory CSF picture, be treated with steroids and harbour a possibility of recurrence.

## Case

A 28-year-old lady of Southern Chinese origin with a history of Behcet’s disease (BD) presented with a new onset of headache. For five years prior, she experienced symptoms of recurrent oral ulcers and ocular findings (panuveitis with cystoid macular oedema). She had been first treated with steroids for four years before they were stopped 11 months prior to presentation in view of bilateral posterior subcapsular cataracts, hypertension and weight gain (weight of 94.5 kg (BMI 37.4) around the time steroids were stopped compared to her baseline of about 70 kg, Table 1). She had been on methotrexate for two and half years before a change to mycophenolate mofetil (MMF) two months prior to presentation. She was also on adalimumab for the last two years. However, her ocular disease (inflammation and oedema) was not well-controlled, suggesting inadequate immunosuppression. Other than calcium and Vitamin D, there was no supplemental medication use including traditional medications.

**Table 1:** Weight and BMI change pre and post-presentation

Date	6/19	1/20	4/20	9/20 *	12/20	4/21	7/21	8/21	4/22	9/22		11/22	7/23
Weight (kg)	89.6	89.6	92.6	94.5	91.4	92.9	88.3	87.2	78.7	79.9		79.6	80.9
BMI	35.4	35.4	36.6	37.4	36.2	36.7	34.9	34.5	31.1	31.6		31.5	32.0

*Legend* 6/19: Refers to the month of June 2019

* Stopped Prednisolone around July 2020

Two weeks prior to current presentation, she had developed gradual onset of holocranial headache, of pressing character, moderate to severe intensity, without postural variation lasting one week which then resolved. There was no nausea or vomiting, new visual disturbances, or other focal neurological deficits. Systemic review was unremarkable for symptoms of infection, sleep apnoea, urinary or bowel disturbances, new arthralgias, orogenital ulcers or recent vaccination. There was no recent weight gain, with her weight actually having dropped 6 kg (BMI now 34.9) over the last nine months (Table 1). At a routine review, asymmetrical disc swelling was detected (optical coherence tomography retinal nerve fibre layer (OCT RNFL) disc thickness 331 μm and 194 μm on right and left respectively) (normal is 102 ± 7 μm) [1]. Humphrey’s visual field testing showed an enlarged blind spot bilaterally with normal confrontational testing. There was a right eye grade 1 relative afferent pupillary defect. Neurological examination was unremarkable with regards to cranial nerves, tone, reflexes, power, sensation, and cerebellar function. Contrasted MRI of the brain, orbit and venogram was normal. There were no radiological features of IIH such as flattened optic discs, tortuous optic nerves, enlarged Meckel’s caves, empty sella or acquired tonsillar ectopia. There were also no orbital or intracranial lesion, enhancement, hydrocephalus, or venous thrombosis. Lumbar puncture (LP) showed an opening pressure > 40 cmH20, 151 leucocytes (mainly lymphocytic) without erythrocytes, glucose 2.7 mmol/L (capillary glucose was 7.4mmol/L) and protein was slightly raised at 0.56 g/L. Cerebrospinal fluid (CSF) screen for bacteria, viruses, tuberculosis, fungi, cryptococci was negative. CSF flow cytometry showed large B cells. CSF cytology did not show malignancy. Serum biochemistry, erythrocyte sedimentation rate, inflammatory markers, angiotensin converting enzyme levels were normal.

The impression was that of a lymphocytic meningitis with severely raised intracranial pressure (ICP), on background of BD. Given uncertain etiology and potential morbidity, the treatment approach was broad. She was covered with antimicrobials until the infective screen returned negative. As an inflammatory, possibly autoimmune process related to her BD could not be ruled out, her immunosuppressive treatment was consolidated with initiation of prednisolone 60 mg OM whilst adalimumab and MMF were held off. Idiopathic intracranial hypertension (IIH) was considered as a differential given significantly elevated pressures and the patient’s body habitus; and she was empirically treated with oral acetazolamide. She was initiated on a dose of 250 mg thrice daily, with plans to up-titrate to IIH treatment dosing of 500 mg twice a day, and even higher if indicated.

A targeted second LP was repeated six days later in view of large B cells in previous CSF flow cytometry. It showed an opening pressure of 36.5 cm H20 and resolution of raised protein (0.29 G/L). The cell count was not rechecked. Flow cytometry did not show clonal proliferation.

After an initial improvement in disc swelling, the prednisolone was downtitrated (refer to Table 2). The acetazolamide had to be stopped due to metabolic acidosis. An LP was once again repeated which confirmed there was no clonal proliferation. Opening pressure was 36.5 cm H20, however there were no elevated CSF leucocytes or protein.

**Table 2:** For illustration of temporal correlation between events, disc thickness and medication dosing

Date M/Y	6/21	7/21	8/21	9/21	10/21	12/ 21	1/22	2/22	3/22	4/22	5/22	6/22	9/22	11/ 22	12/ 22	4/23	7/23
**Event**	Initial adm LP OP > 40 CP 16	LP OP > 36.5 CP 18	-	LP OP > 36.5 CP 16.5	-	-	-	-	-	Rpt adm OP 41 CP 14.5	LP OP → 34.5 (9/7 post CYC) CP 19.5	-	-	-	LP OP 30 CP 20	-	LP OP 31.5 CP 19
**OCT RFNL thickness (µm)**																	
RE	331	180	177	177	171	170	-	170	-	177	-	158	163	-	-	155	-
LE	194	154	149	163	165	155	-	150	-	153	-	142	147	-	-	138	-
**Treat-ments utilised**																	
ACTZ dosing in 250 mg	TDS	TDS	Stop- ped	-	Restarted at BD	BD	BD	TDS	TDS	QDS	QDS	QDS	QDS	QDS	QDS	QDS	TDS
PO PNL OM dosing (in mg)	60	30 → 15	10	10	10	10	8	8	6	IV MP x 3/7, f/by pred 60 mg OM	55→ 35	35→ 30→ 15	10	5	5 EOM	5 EOM	5 EOM
PO MMF	-	-	-	-	500 mg BD	1 g BD	1.5 g BD	1.5 g BD	1.5 g BD	Stopped	-	-	-	-	-	-	-
S/C Adali-mumab 40 mg	-	-	-	-	q 2 weekly	q 2 weekly	q 2 weekly	q 2 weekly	Weekly	Stopped	-	-	-	-	-	-	-
CYC	-	-	-	-	-	-	-	-	-	-	Started	Continued cycle 2	-	Finished 6 cycles	-	-	-
S/C tocilizumab 162 mg weekly	-	-	-	-	-	-	-	-	-	-	-	-	-	Start	Cont	Cont	Cont

M/Y means month/year. Adm: admission. LP OP refers to lumbar puncture opening pressure (in cm H20) whilst CP refers to closing pressure (also in cm H20). Rpt means repeat. 9/7 means 9 days. ACTZ is acetazolamide. PNL is prednisolone. MMF is mycophenolate mofetil. IV cyclophosphamide is intravenous CYC. IV MP x 3/7, f/by pred 60 mg OM stands for ‘Intravenous methylprednisolone for 3 days followed by PO prednisolone 60 mg OM. Cont means continued

The differential diagnosis at this point included an autoimmune cause possibly NeuroBehcet’s (NB)-related inflammatory process and IIH. The diagnosis of IIH was mainly supported by her obese habitus. However, compared to nine months prior to her presentation when her BMI was at its peak and her steroids had been also stopped, at the time point when she eventually presented her BMI had dropped 2.5 points and had been on an overall downward trend. This downward weight trend was not supportive of a new development of IIH). Furthermore, the headache was not prominently associated with the raised ICP, lymphocytic meningitis is inconsistent with IIH, and imaging was not supportive of IIH. Nonetheless she had persistent disc swelling (OCT RFNL 177 and 163 μm) and raised ICP. Given the lack of alternatives, she was re-trialed on acetazolamide at a low dose, which was subsequently increased. She was restarted on adalimumab and MMF which were then uptitrated, whilst prednisolone was weaned off.

Ten months after her initial presentation, she complained of daily headaches, occurring in association with bilateral mildly limited abduction, orogenital ulcers and left eye uveitis, again suggesting inadequate control of the underlying BD. A LP was performed showing an opening pressure was 41 cmH20 but CSF was otherwise bland (protein was 0.33G/L and 2 white cells).

The decision was made to treat her as NeuroBehcet’s-related intracranial hypertension without cerebral venous thrombosis (NBrIHwCVT) given her background of BD, initial lymphocytic meningitis, and lack of response to acetazolamide rather than IIH. Therefore, she was pulsed with steroids, followed by cyclophosphamide whilst non-steroid immunosuppressants were stopped. About a month after the prior LP, she showed an early response to high dose steroids and intravenous cyclophosphamide, with pressure decreasing to 34.5 cmH20. Headache, vision and OCTs (158/142) continued to improve as she was completing her cyclophosphamide with down-titrating prednisolone. However, she still had bilateral active ocular Behcet’s after 6 cycles of cyclophosphamide. After consideration, a novel treatment approach was initiated with weekly subcutaneous Tocilizumab. After a month of tocilizumab, the opening pressure had further declined to 30 cmH20, demonstrating apparent significant response to immunosuppressive therapy, further confirming the diagnosis of NBrIHwCVT. This was associated with gradually decreasing disc swelling OCTRFNL values over the next few months. The ocular BD also showed some improvement with regards to her retrolental inflammation and peripheral vasculitis. After seven months of tocilizumab, the ICP remained similar (31.5 cm H20). The maintenance of ICP at about 30–31 cm H20 even seven months later, and a decrease in OCT RFNL thickness points to efficacy of Tocilizumab in the absence of cyclophosphamide action. However, further attempts to bring down her ICP remains challenging despite trial of multiple immunosuppressant agents. Acetazolamide was also continued however, its effect on the ICP was likely limited given ICP remained unchanged at three times a day dosing.

## Review of the literature

NB is an uncommon manifestation of BD affecting < 10% of patients with the disease [2]. It is generally divided by the presence or absence of parenchymal involvement. Raised ICP in NB generally occurs in association with cerebral venous thrombosis, occurrence without thrombosis is rare [3], as evidenced by limited case reports.

We searched for publications on PubMed and Google Scholar with various combinations of ‘Behcet’, ‘NeuroBehcet’s’, ‘intracranial hypertension’, ‘raised intracranial pressure’ and ‘without cerebral venous thrombosis’. Publications not in English were excluded. Paediatric patients and patients who subsequently developed CVT were excluded. Cases were also excluded if there was uncertainty on adequacy of imaging to exclude CVT as well as no documentation of performed LPs. We identified 14 publications, with 34 patients including ours. Information on demographics, imaging modality, exclusion of parenchymal lesions, CSF pressure, exclusion of CSF inflammation, treatment given and/or outcome was tabulated (Table 3). The majority, barring seven patients, had incomplete data. Of note, there was a recent review of reports from 18 patients inclusive of paediatric cases.

**Table 3:** Summary list of publications with patients with Neuro-Behcet’s related intracranial hypertension without cerebral venous thrombosis

Ref letter	No	Dem	Mod.	Par.	OP	CSF inf	1st pres.	Rx	Otcm	Notes
**A**	1	-	CT/ MRI	N (no mass lesions)	> 25	N	-	-	Freq relapses	2 pts of 16 not adequately inxn for CVT whilst 10 had CVT. 1 had vena cava syndrome. In the IH group thrombophlebitis was a frequent manifestation while uveitis was rather rare. IH in BD has a more benign course than other types of neurologic involvement.
	1	-	CT/ angio	N (no mass lesions)	> 25	N	-	-	Freq relapses	
	1	-	MRI/ angio	N (no mass lesions)	> 25	N	-	-	Freq relapses	
**B**	1	38/F	MRI	N	35.4	N (Subsequent protein raised)	Y	IV MP, PNL, AZT	Recurrence after 1 year. 1st episode not treated with immuno-suppression	
**C**	1	36/F	MRI	N	36	N	Y	IV MP, PNL	CSF pressure normalised	
**D**	1	23/M	Angio	N	Not raised	N	Y	ACTH, Chlorambucil	Remission	CSF pressure was not raised as patient was improving.
	1	32/F	CT	N	32	N	Y	PNL, Chlorambucil	LP drainage. Remission, recurrence after 8 months, then recovery	
**E**	1	30/M	CT	N	> 30	N	Y	IV MP, PNL, AZT	Resolved in 1 month but recurred 6 months later	
	1	36/F	CT	N	> 30	N	Y	IV MP, PNL, AZT	Improved in 1 week, but bilateral optic atrophy remained	
**F**	8	26–57 years, 3 were male, 5 female.	MR	N	> 25	-	-	IV MP, AZT	Resolved	2 of 8 hd CSF TW > 5, although protein normal.
**G**	2	36 M and 47 M	CT/ angio	-	> 24	Protein for each pt uncertain but nil cellular reaction	Y	-	Not specific for those without CVT	
**H**	3	-	CT or MRI	N	Raised	N	-	-		
**I**	1	21 F	-	-	31	N	Y	-	-	No data on whether CVT was present.
**J**	1	32 F	Normal but modality not doc	-	30	Protein 20 mg/dL	-	Steroids	-	Recovery after 4 months
	1	22 M	-		30	Protein 30 mg/dL	-	Steroids bilateral decompression		Secondary optic atrophy after 1 year
**K**	3	-	CT/MRI	-	Raised	-	-	-	-	Had recognized precipitating factors.
**L**	1	-	MR	N	Raised	-	-	-	-	
**M**	1	-	CT	N	Raised	-	-	-	-	
**N**	3	-	Not known	N	-	-	-	-	All 4 cases underwent shunting and remained well	
**O**	1	28 F	MRI	N	> 40	Only on 1st LP, subsequently no evidence of inflammation	Y	Steroids, MMF, adalimumab, CYC, tocilizumab	ICP decrease without normalization	

Ref letter: reference letter. No: refers to number of cases. Dem: demographics, i.e. age and gender. Mod.: refers to imaging modality. Par. : refers to presence of parenchymal lesion. OP: opening pressure. CSF inf. : refers to presence of CSF inflammation. Y: yes. N: no. 1st pres. : First presentation of NB. Rx: treatment. Otcm: outcomes

Notes: Exclusion details and other notes

**References by reference letter(s)**:

A. Akman-Demir G, Bahar S, Baykan-Kurt B, et al. lntracranial hypertension in Behcet’s Disease. Eur J Neurol 1996;3:66–70

B. Lin A H, Chen D Z, Tan C WT. Neuro-Behçet’s disease presenting as isolated intracranial hypertension. Ann Acad Med Singap. 2021 Jan;50(1):88–89

C. Guney F, Bozkurt B, Paksoy Y (2016) Intracranial Hypertension in Behcet Disease: A Case Report. J Clin Case Rep 6: 748

D. Wilkins M R, Gove R I, Roberts S D, Kendall M J. Behçet’s disease presenting as benign intracraknial hypertension. Postgrad Med J. 1986 Jan;62(723):39–41

E. Teh L S et al. (1990). Recurrent papilloedema and early onset optic atrophy in Behçet’s syndrome. Ann Rheum Dis 49: 410–411

F. Akdal G et al. Pseudotumor cerebri syndrome without cerebral venous sinus thrombosis in Behçet’s disease. J Neurol Sci. 2017 Dec 15:383:99–100

G. Shakir R A, Sulaiman K, Kahn R A, Rudwan M. (1990). Neurological Presentation of Neuro-Behçet’s Syndrome: Eur Neurol. 1990;30(5):249 − 53

H. Siva A, Kantarci OH, Saip S, Altintas A, Hamuryudan V, et al. (2001) Behçet’s disease: diagnostic and prognostic aspects of neurological involvement. J Neurol 248: 95–103

I. Cook G, Tagg N T. Idiopathic intracranial hypertension and autonomic neuropathy as the heralding manifestations of neuro-Behcet’s. Neurology. April 18, 2017; 88 (16 Supplement): P5.312

J. Pamir M N, Kansu T, Erbengi A, Zileli T. Papilledema in Behçet’s Syndrome. Arch Neurol. 1981;38(10):643–645

K. Celebisoy N, Seçil Y, Akyürekli O Pseudotumor cerebri: etiological factors, presenting features and prognosis in the western part of Turkey (2002). Acta Neurol Scand 106: 367–370

L. A Al-Araji, K Sharquie, Z Al-Rawi. Prevalence and patterns of neurological involvement in Behcet’s disease: a prospective study from Iraq. J Neurol Neurosurg Psychiatry 2003;74:608–613

M. Al-Fahad S A; Al-Araji A H. Neuro-Behcet’s disease in Iraq: a study of 40 patients. (1999) 170(2), 105–111

N. Kidd D, Steuer A, Denman A M, Rudge P. Neurological complications in Behçet’s syndrome. Brain, Volume 122, Issue 11, Nov 1999, 2183–94

O. Our case which has been presented in this manuscript

Our literature review revealed that the majority of NBrIHwCVT patients were young (third to fourth decade of life, with an average age of 34 years), with a slight female preponderance (12 of 20 patients with available data), in keeping with our patient’s demographics. Interestingly, our review found that in patients for whom we have details of presentation all the patients had presented with NBrIHwCVT as first presentation of NB. We found that of the 17 cases in the literature (not including ours) with available data on CSF profile, none had raised leucocytes whilst one patient had elevated protein; this non-inflammatory CSF profile was similar to our case. A non-inflammatory CSF however, would be contrary to the expected findings for NBrIHwCVT which is thought to be immune-mediated. This may make diagnosis challenging. Our case also highlighted an interesting point, where after the first lumbar puncture, subsequent LPs have all been bland with, however, persistently raised CSF pressures, suggesting that CSF findings may depend on the timing of the LP. The diagnosis of NBrIHwCVT would need to be made assimilating both CSF profile as well as other clinical features, underscoring the challenging diagnostic process.

Patients were generally treated with steroids and occasionally azathioprine, in line with the suspected autoimmune pathophysiology. The use of Tocilizumab, an IL-6 recombinant humanized anti–IL-6 receptor monoclonal antibody was not reported in any of the cases in the literature review. None of the patients were treated with anticoagulation.

Only 22 patients had data on outcome, of which six (27%) were noted to have recurrence of symptoms generally occurring a few months later based on the limited data available. However, there is insufficient data to draw definite conclusions on typical disease course of NBrIHwCVT. Our patient’s disease course would be considered quite refractory and severe, failing to respond to multiple immunosuppressive agents and was unlike the course of BD and NB previously reported in Southern Chinese [4].

## Discussion

As demonstrated by this case, intracranial hypertension secondary to NeuroBehcet’s can present with BD with raised ICP even if there is no prior history of NB, central Asian ethnicity or cerebral venous thrombosis. We demonstrated how novel use of Tocilizumab may have a role in the management of NBrIHwCVT.

The pathophysiology of BD involves alteration of cellular immunity and cytokines, the latter which includes IL-6 [5] which plays an essential role in BD [6] whilst the pathophysiology of NB remains unknown [6]. IL-6 is a multifunctional cytokine involved in immune regulation, hematopoiesis, inflammation, and neural development, and it has a major role in the brain’s response to injury [6]. Liu et al. described that the pro-inflammatory cytokines produced by infiltrating T lymphocytes and monocytes, such as IL-6, might result in neuronal apoptosis [6]. The pathophysiology of NBrIHwCVT is not well understood. It has been suggested that there may be an immune-mediated impairment of CSF re-absorption across arachnoid villi and this explanation is supported by the good clinical response to immunosuppression. Another possibility might be inflammation of the venous sinus wall [7].

For BD-related neurologic disease including manifestation such as meningitis and myelitis and not specifically NBrIHwCVT, Tocilizumab has been described to be an effective alternative to conventional treatment options e.g. anti-TNF agents [8, 9]. Recognized treatment options for parenchymal NB include steroids, azathioprine, anti-TNF-α agents and cyclophosphamide whilst anti-IL6 agents have limited clinical evidence [10]. With regards to treatment options for non-parenchymal BD, we can consider the example of, i.e. BD-related cerebral venous sinus thrombosis. This condition is treated with steroids and anticoagulation acutely whilst azathioprine, cyclophosphamide, cyclosporin and anti-TNF-α agents can be considered in relapsing cases [10]. Lastly, patients with BD with arterial disease as an example of vascular involvement are typically treated with steroids and cyclophosphamide, with small studies reporting improvement with tocilizumab [11]. As such, Tocilizumab may have a potential role in treating raised ICP secondary to NB, increasing treatment options for clinicians in future and it would make our case an important contribution to the literature.

## Data Availability

No datasets were generated or analysed during the current study.
